# ‘It’s just a Band-Aid!’: Public engagement with geoengineering and the politics of the climate crisis

**DOI:** 10.1177/09636625221095353

**Published:** 2022-05-13

**Authors:** António Carvalho, Mariana Riquito

**Affiliations:** University of Coimbra, Portugal; University of Coimbra, Portugal; University of Amsterdam, The Netherlands

**Keywords:** Anthropocene, climate crisis, geoengineering, public engagement with science and technology, Zoom

## Abstract

Geoengineering consists of a set of techniques whose aim is to avoid the disastrous consequences of global warming, such as solar radiation management and carbon dioxide removal. Due to its controversial nature, over the past decade various exercises of public participation with geoengineering have been put in place, particularly in the Global North. This article draws on empirical data stemming from six online focus groups on public engagement with geoengineering conducted in Portugal. In contrast to previous research, we included situated publics to emphasize the political implications of geoengineering, bringing to the debate those with a potential stake in the matter – environmentalists, activists, university students, science communicators and promoters of holistic practices. We suggest that the elusive and uncertain character of geoengineering moves the discussion away from its technological specificity, bringing to the fore some of the socio-political, economic and ethical tensions underpinning the climate crisis.

## 1. Introduction

Geoengineering can be defined as ‘the deliberate large-scale intervention in the Earth’s climate system, in order to moderate global warming’ (Royal Society, 2009: 1), aiming to ‘decouple the climate from humanity’s cumulative emissions of carbon dioxide’ (Morton, 2015: 26). There are two main forms of geoengineering: solar radiation management (SRM) and carbon dioxide removal (CDR). SRM attempts to tackle climate change by altering the Earth’s albedo (Carr et al., 2013), reflecting solar radiation back into space and cooling down the Earth. CDR aims to directly remove CO_2_ from the atmosphere, increasing the capacity of natural or engineered carbon sinks, reducing CO_2_ concentration (IPCC, 2012).

Currently, there are small-scale geoengineering projects underway, including one headed by David Keith: the Stratospheric Controlled Perturbation Experience (SCoPEx). Keith and his colleagues aimed to conduct a series of scientific balloon flights to test the cooling capacity of SRM *in situ* in Sweden in June 2021 (Temple, 2021), but this was cancelled due to opposition from environmentalists, scientists and indigenous communities.^1^ Regarding CDR projects, the Swiss company Climeworks developed a CO_2_ air capture technology able to store it in geological formations. Other geoengineering techniques include bio-energy with carbon capture and storage (BECCS) and afforestation (Waller et al., 2021). BECCS captures and stores CO_2_ resulting from the combustion of biomass, permanently removing it from the atmosphere (Bui et al., 2018). Afforestation projects – such as the Haryana cooperative afforestation project (India) or the Guangxi watershed project (China) – ‘increase the plant and soil sink of atmospheric CO_2_ through photosynthesis’ (Zhang et al., 2015). Other proposals being discussed include the construction of space reflectors or the genetic manipulation of plants to enhance their ability to remove CO_2_ from the atmosphere (Oxford Geoengineering Programme, 2018).

Within scholarly debates on the Anthropocene, geoengineering is often portrayed as a hypermodern ‘technofix’ raising serious ethicopolitical concerns (Szerszynski et al., 2013), namely, the expansion of capitalist – and extractivist – domination into new frontiers: the stratosphere, the oceans and space. Geoengineering can be understood as an attempt to control ‘earth systems in order to combat – or at least to reduce – the negative consequences of capitalist externalization’ (Altvater, 2016: 151), instead of tackling the deep-rooted causes of the climate crisis, entwined with socioeconomic and political systems. Following this line of thought, Szerszynski et al. (2013) argue that geoengineering – and specifically SRM – is an inherently political technology, favouring certain social patterns and power relations, thus jeopardizing democracy. However, Heyward and Rayner (2015) suggest that social scientists are highly critical of geoengineering applications (such as stratospheric aerosol injection (SAI)) ‘at a much earlier stage than they have been of proposals for drastic emissions cuts in the mainstream climate-change discourse’ (p. 102).

There have been attempts over the past decade to involve lay publics specifically in the discussion of geoengineering through focus groups (FGs), interviews and other forms of deliberation (Bellamy et al., 2016; Bellamy and Lezaun, 2015; Buck, 2018; Cox et al., 2020; Pidgeon et al., 2012). The Royal Society (2009) and the IPCC (2012) produced reports on geoengineering, and recently the European Research Council funded a project^2^ on the environmental, technical, social, legal and policy dimensions of SRM and CDR, reinforcing the institutional relevance of this topic.

This article draws on empirical data collected from six online FGs on geoengineering – the first event of the kind conducted in Portugal on this topic. The main research question it addresses concerns the ways in which geoengineering articulates the socio-political, economic and ethical tensions underpinning the climate crisis. We argue that geoengineering discussions foreground particular aspirations and ways of addressing climate change, thus unpacking some of the paradoxes of the Anthropocene, allowing participants to speculate on alternatives to the (hyper)modern acceleration characterizing our planetary zeitgeist (Swyngedouw and Ernstson, 2018). Our FGs explicitly attempted to politicize geoengineering discussions by including environmentalists, university students, activists, science communicators and promoters of alternative therapies. In doing so, we respond to Nowotny’s (*apud* Irwin, 2014: 75) call to ‘bring the political back’ into public engagement exercises with emerging technologies. Our FGs led to debates on the manifold contradictions of the climate crisis, including North/South unbalances, socioeconomic disparities and uncertain risks stemming from technologies in the making.

This article is organized as follows: the literature review presents an overview of research on public engagement with geoengineering, emphasizing our scholarly contribution. The methodological section focusses on the organization, composition and practical aspects of the FGs. The analysis delves into the main themes cutting across the FGs: (1) unexpected risks; (2) uncertainty and unfamiliarity; (3) geoengineering as a Band-Aid; (4) as a way to perpetuate global inequalities; (5) as an expansion of the capitalist-extractivist paradigm; (6) as a climate justice matter and (7) geoengineering within the Portuguese context. The discussion focusses on the experimental dimension of our FGs, the participants’ and our own situatedness, the importance of the Portuguese context and the articulation of knowledge and ignorance. The conclusion explores some of the limitations of our study, suggesting directions for future research.

## 2. Public engagement with geoengineering

Upstream engagement with emerging technologies – such as nanotechnologies, synthetic biology and geoengineering – is key to problematizing their potential social, political and ethical implications (Anderson et al., 2008). Geoengineering, due its emergent and controversial nature, is considered ‘an ideal case for [conducting] upstream public engagement’ (Wibeck et al., 2017: 3).

Nonetheless, despite its contentious character, public engagement with geoengineering is still scarce (Bellamy et al., 2017; Bellamy and Lezaun, 2015). In 2018, there were only ‘around 30 empirical social science studies on public perceptions of climate engineering’ (Buck, 2018: 79). Although the large majority treats geoengineering as a ‘global object’, instead of focussing on a specific technique (Buck, 2018; Wibeck et al., 2017), some exercises have delved into specific applications. Corner et al. (2011) carried out public engagement with three CDR ideas and two SRM applications; Macnaghten and Szerszynski (2013) looked at SAI, the most famous SRM proposal; Bellamy et al. (2016) looked at two CDR techniques and one SRM application; Bellamy et al. (2017) looked at two CDR and two SRM ideas; and Cox et al. (2020) analysed the public perceptions of three CDR ideas.

This body of research remains geographically limited, as most studies have been conducted in the Global North. The only exceptions to this are Winickoff et al. (2015), Carr (2015), and Whyte (2018). Carr (2015) specifically addressed the perspectives of citizens in Kenya, the Solomon Islands and Alaska Natives and, alongside Whyte (2018), he is the only author who has conducted public engagement with indigenous communities. Hence, research on potential ethical and socio-political aspects of geoengineering often excludes the Global South, even though these populations will be the most affected both by climate change and potential geoengineering technologies (Winickoff et al., 2015).

Despite the different geographical contexts or applications being evaluated, participants often express common views and share similar concerns regarding geoengineering (Burns et al., 2016; Carr, 2015; Corner et al., 2012; Merk et al., 2015). Wibeck et al. (2017), who organized FGs with 136 participants from Japan, New Zealand, the United States and Sweden, noted that common themes emerged across them all. This is usually the case for technologies like geoengineering because people tend to use similar mind schemes to make sense of novel and emerging technologies, which are not yet subject to entrenched social representations (Rogers-Hayden and Pidgeon, 2007; Wibeck et al., 2017). Participants often craft their perceptions of geoengineering according to its perceived degree of controllability, although controllability itself is multi-faceted (Bellamy et al., 2017).

Drawing on previous literature reviews (Burns et al., 2016; Corner et al., 2012; Merk et al., 2015) and on our own review of existing scholarship, we identify four main recurring patterns within public perception of geoengineering: (1) general unfamiliarity; (2) its general perception as something risky; (3) geoengineering as a last-resort solution, often related to the ‘moral hazard’ hypothesis and (4) a preference for other climate policies.

The first tendency is the general unfamiliarity with geoengineering. There is limited public knowledge about these technologies: according to Mercer et al. (2011), who conducted the first large-scale international survey of public perception of geoengineering in 2010 in the United States, Canada and the United Kingdom, only 8% of respondents could define ‘geoengineering’. Merk et al. (2015) confirmed this tendency in Germany, noting that public awareness of geoengineering ‘remains low’ (p. 300). Hence, ignorance plays a significant role in lay discussions. Participants often use analogies or compare geoengineering with more familiar technologies, emphasizing their ignorance and unease (Wibeck et al., 2017), arguing that research seems uncertain, doubting whether scientists will ever have enough knowledge to justify these interventions (Burns et al., 2016; Mercer et al., 2011; Wibeck et al., 2015).

The second pattern concerns the public perception of geoengineering as something risky. Merk et al. (2015) found that 81% of respondents in Germany viewed the *overall risk* of geoengineering as ‘very large’ or ‘somewhat large’, and Wibeck et al. (2017) stated that the large majority of their research participants reacted with fear and anxiety to geoengineering, displaying concerns regarding the loss of human control and potentially risky consequences. Risk perception is also affected by particular applications. In Mercer et al. (2011) survey, CDR was usually favoured over SRM. CDR’s technologies are often understood as ‘more natural’ in part because FGs’ facilitators unintentionally portrait them as such. The artificialness/naturalness dualism is one of the main factors influencing the public perception of these technologies (Burns et al., 2016; Corner et al., 2012).

The third tendency is the ‘moral hazard’ hypothesis – the idea that geoengineering will lead to a dis-responsibility at both the individual and political levels (Burns et al., 2016; Corner et al., 2012; Fairbrother, 2016; Winickoff et al., 2015). Research participants often categorize geoengineering as a ‘last resort solution’, viewing it as a ‘dangerous distraction’ rather than a solution to the climate crisis (Buck, 2018; Burns et al., 2016; Wibeck et al., 2017). The moral hazard hypothesis has been the strongest argument against geoengineering, emphasizing shared concerns among heterogeneous lay publics (Fairbrother, 2016; Winickoff et al., 2015).

The fourth pattern derives from the latter: citizens generally tend to prefer other responses to the climate crisis – either mitigation policies (Burns et al., 2016) and/or political and individual changes (Wibeck et al., 2017) – to the detriment of geoengineering. It is often argued that geoengineering would reproduce the same existing patterns, and participants normally favour changes in lifestyles or global political policies. Moreover, when contrasting geoengineering with mitigation policies, participants often consider the latter as more urgent and timely than technological solutions.

Merk et al. (2015) have shown that the acceptance of geoengineering was associated with the belief that climate change is a serious problem. Similarly, Braman et al. (2012) found that learning about geoengineering increases participants’ risk perception of climate change. A crucial influencing factor on the acceptability of geoengineering is the trust in science and technology. Macnaghten and Szerszynski (2013) categorized five general conditions for public acceptance of geoengineering: scientific robustness; research foreseeability; research efficacy; effective governance and democratic condition, including trust in experts and concerns about transparency and democratic accountability.

Although geoengineering has sparked a lot of debates over the past decade, little research has been conducted with situated publics, that is, those with potentially robust stances on the socio-political implications of these technologies due to their political, epistemological or social position. Moreover, as Buck (2018) suggests, most research has not examined the public perception of geoengineering in the context of a particular place, disregarding how situated cultural knowledge, traditions and history might shape the public perception of these technologies. Our study aims to address these research gaps, by engaging situated publics, namely, environmentalists, activists, university students, science communicators, and practitioners of alternative therapies, as well as attempting to situate the potential implications of geoengineering according to the particularities of the Portuguese context, exploring how socio-political and geographical situatedness can foster particular ethical and political stances.

## 3. Methodology

Due to the coronavirus disease (COVID)-19 pandemic, we had to conduct the FGs online, through Zoom. Over the course of 2 weeks, in December 2020, we conducted six FGs. Each discussion lasted between 1 and 2 hours, considered to be the ideal time for well-designed FGs (Onwuegbuzie et al., 2009). All sessions were fully recorded, transcribed; participants were anonymized, and they signed an informed consent form.

The FGs included different profiles to evaluate similarities and differences between divergent backgrounds. All groups were gender balanced. Our politically situated approach followed previous exercises of public engagement with emerging technologies conducted by our research centre. Group I was composed of seven environmentalists, belonging to different environmental nongovernmental organizations, social movements or grassroots ecological organizations, and should ideally display strong stances on the climate crisis. Since climate change brings to the fore issues of intergenerational justice, Group II was composed of seven university students studying different subjects at different levels of education (from Bachelor to PhD). Group III gathered five activists – from feminist and LGBTQIA+^3^ organizations, animal rights collectives, trade unions and student movements – and it was expected that they would be concerned with socioeconomic and political implications of geoengineering as well as its relationship with other causes. Group IV included seven science communicators from fields related to geoengineering: Earth Sciences, Climate Sciences, Geostatistics and Volcanology. They were expected to display a positive view on scientific and technological progress and to be more aware and knowledgeable of this topic than the others. Group V was only composed of two members (due to unexpected drop-outs) engaged in alternative and holistic therapies and practices. It mirrored the previous one, as these participants were expected to have a sceptical and critical view of modern science and technology. Group VI was heterogeneous, supposed to represent the broader ‘Portuguese lay public’, and included six participants with different professional statuses, ages and genders, and from different cities.

Instead of working with a disengaged or abstract notion of ‘public’, we included participants whose background, affiliation, situationality and/or socio-political involvement would generate strong political stances on geoengineering. Hence, the FGs were designed not as an attempt to represent a ‘broader public’ but rather to politicize the discussion. Group VI was thought of as a ‘control group’, meant to test our argument, allowing us to reflect on the merits and limitations of our research design. Our research was informed by Bellamy et al.’s (2017) experimental approach, which attempted to develop alternatives to mainstream forms of deliberation. By engaging situated publics, we explicitly attempted to bring to the fore concerns directly linked to the situatedness of each group: climate and environmental justice (Group I); intergenerational justice (Group II); social justice (Group III); science, technology and democracy (Group IV) and deep ecology (Group V).

Participants were told that they would participate in FGs about emerging technologies to face the climate crisis, except for Group VI, who was told they would participate in a FG on the climate crisis. At the beginning of the sessions, we mentioned the aims of our research project and allowed participants to present themselves. Subsequently, we shared a Power-Point^4^: it started by briefly contextualizing the climate crisis, then presented the definition of geoengineering and its two main techniques – CDR and SRM, resorting to examples of ongoing projects.^5^ Finally, it mentioned some potential advantages and disadvantages of geoengineering, and future applications under debate. The debate was then initiated: although we had a script, we allowed for new questions and comments to arise according to the discussions.

We took notes during the FGs and later exchanged ideas on the general tendencies common to all the groups. We then read the full transcripts and carried out a process of thematic analysis, initially identifying five main themes: unexpected risks; geoengineering as a Band-Aid; uncertainty and unfamiliarity; social and political issues; geoengineering within a broader technological framework. Later, we refined the coding process, identifying seven themes that will be unpacked in the following section.

## 4. Analysis

**Figure 1.**
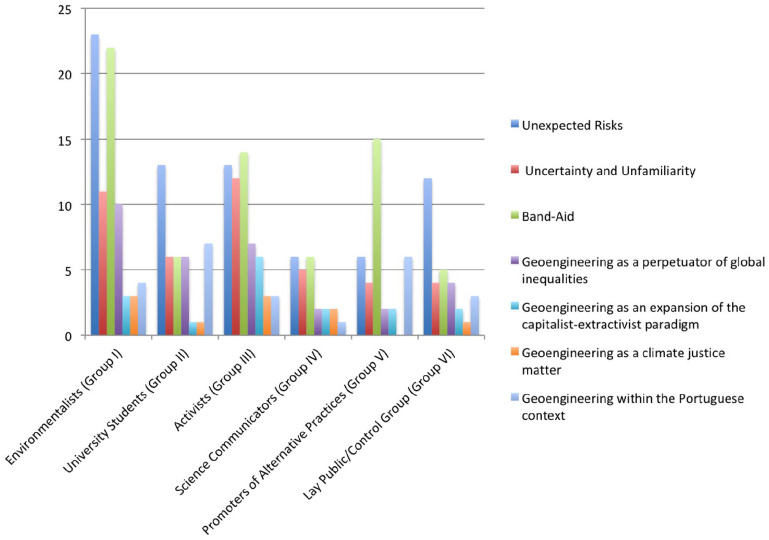
Frequency of debate topics in each group, by number of occurrences.

This section explores the seven main themes underpinning participants’ stances on geoengineering. The first two are somewhat ‘standard’ and are aligned with previous public engagement exercises with geoengineering: (1) unexpected risks (planetary, environmental, health and moral) and (2) uncertainty and unfamiliarity regarding the meaning(s) of geoengineering and its potential effects. The other five themes are a direct result of our situationality-sensitive and culturally-situated research design: (3) geoengineering as a Band-Aid; (4) geoengineering as a perpetuator of global inequalities; (5) geoengineering as an expansion of the capitalist-extractivist paradigm; (6) geoengineering as a climate justice matter and (7) geoengineering within the culturally situated Portuguese context. The following section will unpack these themes by resorting to excerpts from FG transcripts (Figure 1).

### Unexpected risks

When participants discussed potential risks stemming from geoengineering, they mentioned how difficult it was to predict exactly what would happen, stressing potentially unexpected consequences, undermining its large-scale implementation:

We have no idea what that would imply. (BC – group I)

The consequences of these techniques are so hard to assess and quantify (. . .) I see great difficulties in their operationalization in the next few decades. (SMP – group IV)

Participants considered that geoengineering was highly unpredictable and controversial, all techniques displaying potentially negative consequences:

All techniques have negative impacts. (. . .) We are reflecting [referring to SAI], but reflecting where to? What is going to happen outside. . . our planet? All the techniques are controversial because they might have consequences that we have no idea about, so, unless it’s very well studied or event tested on microsystems, it can have very dangerous repercussions. (MF – group II)

While discussing specific applications, there were concerns with the impact of CDR on the oceans, in particular techniques that would involve genetic manipulation of plants:

This idea of manipulating oceans brings a change of ecosystems that may have unpredictable consequences for biodiversity. We’re talking about nature, so the consequences for biodiversity are unpredictable . . . In theory, this is interesting, but I’m afraid its practical application will have consequences and impacts on biodiversity that we are not predicting. (TSNC – group III)

Participants also displayed some concerns with the chemicals involved in SRM applications, questioning whether they could aggravate environmental issues:

We’re polluting our atmosphere with . . . was it sulphur? I’m concerned with the impacts of interfering with our atmosphere in such a drastic way. (CR – group II)

Beyond environmental risks and loss of control, participants suggested that by interfering with Earth Systems there could be unwanted consequences on human health:

Aerosols could cause breathing problems, which would affect our physical and mental health. (SA – group VI)

When we change our atmosphere, it will have brutal effects on our health. (VM – group V)

Some participants even argued that geoengineering posed an existential threat, altering Humanity and its engagement with Nature, permanently interfering with the human condition:

It’s inevitable that these technologies will have an influence on our eating habits, our ways of thinking, our ways of communicating with nature . . . It’s very concerning. (MP – group V)

The disruptive characteristics of geoengineering also entail moral hazards. For some, geoengineering meant that climate change would be decoupled from wider socioeconomic systems, arguing that, when climate change is tackled as a technological issue, it undervalues individual and collective responsibility:

Geoengineering can leave us very passive and conformed, because we think there will always be a solution developed by the scientific community. (FT – group III)

Suddenly, there’s this effect of . . . we go back and can keep doing all the garbage we’re doing because there’s always an alternative. (SC – group VI)

Participants often mentioned the need to develop an ethical framework tailored to the manifold implications of geoengineering:

Genetic issues are dealt with through Bioethics. So it would be interesting to develop an ethics tailored to these contexts. (BC – group I)

The same way that bioethics emerged due to the manipulation of living systems, I think there are also ethical issues linked to these questions. (ASM – Group IV)

### Uncertainty and unfamiliarity

While asking participants about the potential impacts of geoengineering, the most recurring response was ‘I don’t know’. Indeed, the vast majority was hearing about the topic for the first time:

I don’t have an opinion, since this topic is new for me. (AR – Group III)

I don’t know anything about geoengineering. It’s a new topic for me. (VM – Group V)

Even though we provided participants with definitions and mentioned ongoing and future projects, the concept of ‘geoengineering’ was disputed, leading to a wider debate about the climate crisis, often implying that geoengineering became an umbrella term to characterize technological responses to climate change. The discussion, instead of focussing exclusively on the specificities of geoengineering, framed these applications within a wider context coupling concerns with the economy, technology, society and ethics, and participants often shared their struggles regarding the meaning(s) and definition(s) of geoengineering:

I would need a clarification about what we’re talking about . . . I have some troubles debating geoengineering when I don’t know the science very well. (DM – Group I)

I feel that the notion of geoengineering itself is at stake. (. . .) What are we talking about when we talk about geoengineering? (ASM – Group IV)

Public engagement with geoengineering allows citizens to debate on an emerging technology whose controversial applications are still in the making and, although geoengineering is attaining visibility in popular culture, participants were not familiar with it. When we asked them to identify potential risks, they resorted to the lack of knowledge and information to justify their inability to provide us with clear answers:

I don’t know what kind of impact this would have without knowing more about geoengineering. (CR – Group II)

I have no idea . . .That [impact] must be researched . . . [The impact] on people’s health, the balance between ecosystems . . . I don’t know. (MP – Group V)

The lack of knowledge hindered the identification of potential impacts. This was explicitly assumed by most participants, eliciting the difficulties in defining geoengineering and unpacking how these techniques would play out in practice. Consequently, uncertainty was a recurrent theme, affecting participants’ ability to define geoengineering and to identify potential social, ethical, political, environmental and health impacts.

### Geoengineering as a Band-Aid

The third major theme was the representation of geoengineering as a Band-Aid. This metaphorical image was widely used across the FGs:

It’s not a solution, it’s just a Band-Aid! (GL – Group III)

Geoengineering can be sort of a Band-Aid. (FT – Group III)

[Geoengineering] is a Band-Aid, a rapid solution for very profound problems. (VM – Group V)

Participants argued that geoengineering would mask the ‘real causes’ of the climate crisis – for some, the socioeconomic and political system; for others, individual behaviours; for some others, the separation between the human and the non-human world. Participants explicitly voiced that geoengineering would deviate attention from ‘real solutions’, portraying it as a ‘techno fix’ that could never solve environmental and climate issues. As put by some participants, who voiced ecofeminist perspectives, geoengineering reproduces the same techno-scientific paradigm of dominating nature. For them, tackling climate change requires an abolition of the idea of separation between Humanity and Nature:

With geoengineering we are giving priority to a paradigm of control and intervention over nature. (HC – Group I)

[Geoengineering] would perpetuate something that has already been shown not to be sustainable as humanity (. . .) we are going to invest in something that once again represents dissociation between us and nature. (MP – Group V)

Other participants contrasted geoengineering with two distinct responses to climate change: individual lifestyle changes, and the need to transform (or even to abolish) the current socioeconomic system. We noticed a clash between two distinct political ecologies to address climate change, namely, within Group I (environmentalists) and Group III (activists). On one hand, some highlighted how climate change was entwined with individual choices, including consumption patterns, eating habits and recycling:

We are still in a very consumerist society. Until we turn that around, we’ll keep talking about geoengineering. (GA – Group IV)

Change can only be individual. (MP – Group V)

We need to keep alerting people to reduce their ecological footprint. (PE – Group VI)

The solution is by changing how we eat, how we dress . . . (GL – Group III)

On the other hand, some participants identified broader political structures and socioeconomic system as the main culprit of climate change:

We should blame those who produce so much carbon and negatively impact the environment, and it’s certainly not the average citizen (DG, Group II)

We need to go after large corporations to radically change this model . . . of accumulation and intensive extraction of resources. (RC – Group IV)

Eventually, the solution is to change the economic model. (BE – Group VI)

Participants framed geoengineering as a Band-Aid masking underlying issues, namely, individual lifestyles, the current economic and political paradigm, and how it engages (in a deeply violent way) with Nature. Regardless of the political ecologies displayed by participants (whether tending towards lifestyle behaviours or centred on socioeconomic and political aspects), geoengineering was portrayed as a technological fix to tackle a civilizational issue.

### Geoengineering as perpetuating global inequalities

Due to participants’ situatedness, they often mentioned that geoengineering would aggravate existing social and political cleavages, namely, the Global North/South divide and the rich/poor gap. Climate change affects disproportionately the Global South, and disadvantaged populations, while not taking part in the carbon economy, are particularly vulnerable to its consequences. Groups I (environmentalists), II (university students) and III (activists) were particularly vocal about the potential devastating social and political impacts of geoengineering on the Global South:

In regards to North/South inequalities, one of the potential consequences of introducing aerosols into the stratosphere is a reduction of the global average precipitation. But which countries would suffer the immediate and most serious impacts on their economies and agriculture? It would be the South Asian countries, extremely poor countries . . . These would be the first to suffer severe consequences . . . (DM – Group I)

All those machines, those technologies, they’re made of lithium, right? With gases and metals obtained from the Global South. So, it’s that old question again: we’re helping the environment, but at what cost? (RC – Group II)

Probably, the application of these technologies on a global scale will have an impact on North/South relations and worsen the situation of poorer countries already subjected to policy conditionality. (SA – Group II)

Furthermore, and as a direct consequence of North/South inequalities, participants stressed the risk of increasing social inequalities:

These new technologies would create even more power asymmetries. (RC – Group III)

Let us suppose one of geoengineering projects goes wrong . . . Who is better equipped to protect himself/herself from this? It is not those who are more affected by it . . . Undoubtedly, those who would pay the prize are those who have no capacity to do so and are already in a very vulnerable position. (SD – Group I)

In some groups, participants mentioned the risk of the capitalist co-option of these techniques, that is, turning them into a business instead of being used to tackle climate change:

I think this [geoengineering] will easily become another business, overpowering the common good . . . (TSNC – Group III)

The system itself can take advantage from its [geoengineering’s] benefits, turning it into a business. That is capitalism. (FT – Group III)

It’s the economy, it’s money. They [the big corporations] will never stop profiting from the money that the sale of these technologies can generate. (LMA – Group VI)

Geoengineering is understood as ‘business as usual’, capitalism by other means, aggravating existing social and political cleavages, namely, North/South imbalances and wealth gaps. Their potential impacts cannot be decoupled from wider socioeconomic and geopolitical systems, suggesting that geoengineering encapsulates numerous tensions underpinning the politics of the climate crisis.

### Geoengineering as an expansion of the capitalist-extractivist paradigm

While participants emphasized the role of broader political and economic changes, often entwined with a critique of capitalism, there were also concerns that geoengineering could legitimize – and accelerate – extractivism, illustrating a wider paradigm of engaging with Nature that should be questioned:

These new solutions will keep showing up and Nature will keep laughing at us, because we keep moving away from her, when all answers lie in Nature. (PF – Group I)

[With geoengineering] we keep insisting on the same paradigm . . . We’re like children playing Monopoly . . . Completely irresponsible towards Nature . . . Nature isn’t something we can manipulate according to our will! (. . .) We need a paradigm shift. (MP – Group V)

Participants considered that geoengineering would maximize extractivism, with a grim outcome for countries historically affected by colonialism and exploitation, reproducing an unbalanced relation with the environment:

Historically, developed countries have intervened over the Earth and shaped the global territory, without caring about Earth’s ecosystems, our common legacy. (SMP – Group IV)

Who has the legitimacy and where is the science located? Where are the financial means to fund these projects? There are power asymmetries that will be created due to the implementation of these new technologies. (AR – Group III)

### Geoengineering as a climate justice matter

By voicing these concerns, participants portrayed geoengineering as a climate justice matter. Geoengineering was seen as a topic that will be part of future conversations and struggles within the climate justice movement, and social movements more broadly:

This is obviously an issue that has to do with justice! (SMP – Group IV)

There will be a political struggle at this level if the impacts [of geoengineering] prove to be more negative than positive. (FT – Group III)

Participants were concerned that geoengineering could hinder a just transition, keeping the same exploitative socioeconomic system in place, emphasizing the need to take into account the Global South, local communities and indigenous populations:

Who is going to pay for this solution? And who will benefit from this? There is only a fair answer: if those who pay are the ones responsible for bringing us here, and those who benefit are the ones who have been most affected historically. From what I can tell, geoengineering fails to address these two aspects to be a fair solution. (SD – Group I)

Who would support this kind of initiative? Stakeholders such as local communities and indigenous people are ignored most of the time when making these decisions . . . And they are surely against it. (HC – Group I)

Some participants argued in favour of fairer solutions, suggesting the same investment being made in geoengineering should instead be applied to address socioeconomic inequalities at the heart of the climate crisis:

The same investment being made in Geoengineering should be made in social justice. (VM – Group V)

Others advocated for robust and widespread exercises of public engagement, allowing lay citizens to have a say in the development of these techniques:

We need to create debates and accessible conversations for everyone to clarify these concepts, so that everyone can understand proposals such as geoengineering, so that everyone can take part in the debate. (HC – Group I)

When we’re talking about geoengineering, it’s very important to find ways to reach the public, giving them the correct scientific examples (. . .) so that they understand what we’re talking about and can form an opinion on the topic (GA – Group IV)

It is important to develop a broader public debate. Not only at the political level, because those always have the same discourse, but with other discourses and visions, so that other solutions come up. (MP – Group V)

### Geoengineering within the Portuguese context

Participants resorted to specific sociotechnical and environmental controversies in the Portuguese context, such as plastic pollution, lithium mining, nuclear energy, hydroelectric dams and even 5G to argue that climate change could be used as a justification to disregard the negative impacts of geoengineering:

When recycling appeared in Portugal, suddenly there was an increase in plastic consumption, because people felt less responsible – ‘we can use all the plastic we want, then we’ll recycle it’. (TSNC – Group III)

Here in Portugal, we see the issue of lithium. Car batteries have lithium (. . .). We are reducing carbon dioxide emissions at the cost of destroying natural and archaeological heritage, and ecosystems. This also happens with solar panels that contain lead. (BE – Group VI)

It’s a bit like what was done in Portugal with the dams (. . .) nobody remembers that 70% of [renewable energies] is from dams that destroyed countless ecosystems, that caused a drastic decline in the water quality in all the rivers. (BE – Group VI)

Participants relied on the aforementioned sociotechnical controversies to highlight how, while being promoted as a potential solution to climate change, geoengineering could cause a wide range of negative consequences. Moreover, their previous knowledge of national controversies allowed them to recognize that the political elite and the media were often silent regarding previous (or ongoing) potentially disastrous technological projects:

The nuclear station of Almeria is causing damage. This is not being reported by the media. It’s concerning, because it’s really close to us. (VM – Group V)

5G is something that people don’t know much about and has to do with manipulations. It has pros and cons, but people don’t really know what it is. (AR – Group III)

Finally, participants seemed extremely concerned with the application of geoengineering in Portugal due to its semi-peripheral economic-political status and as one of the most vulnerable European countries to climate change. They considered that geoengineering could impact Portugal’s water and soil resources and increase its exposure to extreme weather events, leaving its population in an even worse position:

One of the main environmental problems in Portugal concerns water resources. Interventions in the stratosphere, or wherever, probably would have consequences on the water cycle. (HC – Group I)

What worries me in the case of Portugal is, on the one hand, being a very vulnerable country and, on the other hand, being a country with a reduced economic capacity to invest in geoengineering research . . . So, it is twice as vulnerable. (SA – Group VI)

Nonetheless, it is worth highlighting that these concerns, although emphasizing Portuguese examples and its situationality, didn’t come from a ‘Not In My Backyard!’ position, as research participants voiced concerns indicating how their stances are not determined by nationality but rather by (global) social justice parameters.

## 5. Discussion

Our focus groups were an attempt to elicit situated political stances on geoengineering, drawing on the potential of certain group profiles to discuss the sociopolitical impacts of these technologies. Bellamy et al. (2017) organized experimental deliberative workshops entailing three different formats: majoritarian; consensual; and individualistic, in contrast to dominant forms of public engagement that privilege the ‘egalitarian-consensual model derived from theories of deliberative democracy’ (196). In our case, instead of working with a disengaged notion of ‘public’, we explicitly valued the particular situationalities of participants, assuming that these could strengthen – instead of hindering – geoengineering discussions, emphasizing the role of research design and social sciences’ interventions in the construction of ‘publics’ and in framing (and unframing) debates about this topic (Bellamy and Lezaun, 2015).

As we have seen in the literature review, public engagement with geoengineering often elicits four main topics dealing with unfamiliarity, its risky dimension, the moral hazard hypothesis, and a preference for political solutions (Buck, 2018; Burns et al., 2016; Corner et al., 2012; Merk et al., 2015; Wibeck et al., 2017). The first two main themes we have identified are common while debating this topic and they were also shared within Group VI, the ‘control group’. However, the other five themes we identified emphasize the social and political dimensions of geoengineering, and were often framed by resorting to concerns with environmental, climate and intergenerational justice, deep ecology, ecofeminism, North/South imbalances and socioeconomic inequalities. Some of these topics are the flagship concerns of each situated group, indicating that their group identity and situationality mediated the emergence of sociopolitical concerns with geoengineering, entwining these technologies with issues such as global inequalities, extractivist capitalism, climate justice and the Portuguese context.

Our research design was an explicit attempt to ‘politicize’ public engagement with geoengineering, in light of social sciences’ concerns with some of the most controversial applications, such as SAI (Szerszynski et al., 2013). In that sense, the way we conceived the ‘politics’ of geoengineering was also informed by our own situatedness as ‘critical’ social scientists. The set of materials supporting the FGs, as well as our interventions, confirms the critique of Heyward and Rayner (2015), since social scientists are often more concerned with the sociopolitical impacts of ‘technofixes’ than with top-down climate politics. Our situatedness was also mirrored by the focus groups’ participants as in most groups a wide array of sociopolitical solutions – at the individual, local, national and global levels – were often preferred to the more controversial geoengineering applications that often became the synonym of geoengineering. In fact, although proposals such as afforestation are often understood as forms of geoengineering, our presentation and discussion highlighted the most spectacular ones, inevitably framing geoengineering as a ‘technofix’.

Our research also confirms Braman et al.’s (2012) argument that learning about geoengineering increases the risk perception of climate change. In the case of the FGs, geoengineering became an umbrella term that allowed participants to discuss the climate crisis and its politics. Geoengineering thus becomes politics by other means, a driver to unpack some of the tensions and contradictions of the Anthropocene, allowing participants to debate a wide range of issues inevitably entangled with the climate crisis.

Most research on public engagement with geoengineering is not concerned with the ways in which these technologies are perceived in the context of a particular place (Buck, 2018). Since our FGs were the first exercise of the kind in Portugal, we attempted to explore how our national context could mediate the emergence of sociopolitical implications of these technologies, resorting to questions explicitly focussed on our country. The Portuguese context – including particular sociotechnical and environmental controversies – inevitably shaped the discourse on geoengineering. Some of these issues include lithium mining, Spanish nuclear power plants, dams and 5G. Since geoengineering is perceived as a ‘technofix’, it is framed as representing a continuation of undemocratic and top-down ways of governing the environment, potentially leading to negative health and environmental consequences and increasing North/South inequalities, reinforcing Portugal’s semi-peripheral condition in the European and global contexts.

Finally, a relevant aspect concerns the articulation of knowledge and ignorance. As previous literature has shown, upstream engagement with geoengineering is particularly problematic because some of the most controversial applications are still in the making and involve a high level of uncertainty (Burns et al., 2016; Mercer et al., 2011; Wibeck et al., 2015). Historically, some exercises of public engagement with geoengineering have included experts, supporting the ‘lay public’ in the identification of social and ethical concerns (Bellamy and Lezaun, 2015). In the case of our FGs, and although Group IV included academics with pre-existing knowledge on geoengineering, we didn’t explicitly invite experts to support us in the preparation of these exercises. As social scientists, it could be argued that we are also members of the ‘lay public’, and our own ignorance could have contributed to a caricatural version of geoengineering. As the FGs approached geoengineering as a whole, instead of focussing on specific applications, it became a proxy to unpack the socio-political, economic and ethical tensions entwined with the climate crisis.

## 6. Conclusion

Our article delved into empirical data stemming from online focus groups on geoengineering conducted in Portugal, in December 2020. Our argument is that geoengineering debates are ways of articulating the socio-political, economic and ethical tensions underpinning the politics of the climate crisis.

To unpack the politics of geoengineering, we explicitly engaged with situated publics, inviting environmentalists, university students, activists, science communicators, and promoters of alternative practices, and emphasized some of the particularities of the Portuguese context. Instead of focussing on specific applications, we worked with a broader conceptualization of geoengineering. We relied on participants’ situatedness to trigger a wider discussion about the climate crisis, involving manifold political ecologies encompassing individual, socioeconomic and epistemological aspects. This broad – even elusive – conceptualization of geoengineering allowed us to discuss some of the tensions and ambiguities that characterize the Anthropocene, turning geoengineering into a way of articulating the socio-political, economic and ethical tensions underpinning the climate crisis and its politics.

Some of the limitations of our research include the fact that we, as social scientists, have sparse knowledge on the more technical aspects of geoengineering, which conditioned our ability to clarify some doubts posed by participants. Since the FGs were conducted online, this automatically affected how participants interacted. By creating certain group identities, we inevitably conditioned how the ‘politics’ of geoengineering could be unpacked. This also could have created some invisibilities: for instance, to explore North/South inequalities, we could have invited participants from the Global South, and, although we attempted to invite situated publics, all of them were white and the majority had university level education. Although, through our interventions as social scientists, we attempted to ‘politicize’ public engagement with geoengineering it is possible that, by replicating our FGs with the same research design, we could have obtained a different outcome.

As directions for future research, more work is needed on the impact of the COVID-19 pandemic and digital technologies on public engagement with S&T, and on geoengineering in particular, ideally involving various applications and national contexts. Since geoengineering often blurs the boundaries between science and fiction, it would be interesting to develop exercises focussed on future scenarios involving geoengineering applications. As our research was an attempt to ‘politicize’ geoengineering debates, other situated groups could be involved to assess exactly how their situationality could pave the way for the emergence of certain ethicopolitical stances. In light of Bellamy et al.’s (2017) critique, and to develop alternatives to dominant deliberative models, it would be interesting to experiment with performative and theatrical methodologies, allowing social scientists to assess how these participatory assemblages could shape the public debate on geoengineering. Finally, and recognizing our own limitations as social scientists, interdisciplinary collaborations should be fostered to strengthen public engagement with these technologies.

## Supplemental Material

sj-pdf-1-pus-10.1177_09636625221095353 – Supplemental material for ‘It’s just a Band-Aid!’: Public engagement with geoengineering and the politics of the climate crisis Click here for additional data file. Supplemental material, sj-pdf-1-pus-10.1177_09636625221095353 for ‘It’s just a Band-Aid!’: Public engagement with geoengineering and the politics of the climate crisis by António Carvalho and Mariana Riquito in Public Understanding of Science

## Notes

1.
https://www.nytimes.com/2021/04/02/climate/solar-geoengineering-block-sunlight.html

2. ‘GENIE: GeoEngineering and NegatIve Emissions pathways in Europe’

3. We use the initialism ‘LGBTQIA +’ to refer to Lesbian (L), Gay (G), Bisexual (B), Transgender (T), Queer (Q), Intersexual (I), Asexual (A) people and all others (+) whose sexuality or gender identity is non-normative.

4. This Power Point presentation (in its original, Portuguese version) has been provided as supplemental data.

5. Ongoing projects mentioned in the Power Point presentation include: David Keith’s SCoPEx, the Ocean Iron Fertilization Program of the GEOMAR Helmholtz Centre for Ocean Research and Climeworks’ CDR project.
