# Supplementary material for: ‘It’s just a Band-Aid!’: Public engagement with geoengineering and the politics of the climate crisis
Source: Public Underst Sci. 2022 May 13;31(7):903–20. doi: 10.1177/09636625221095353 (PMC9535969; doi:10.1177/09636625221095353)
Supplement: sj-pdf-1-pus-10.1177_09636625221095353 – Supplemental material for ‘It’s just a Band-Aid!’: Public engagement with geoengineering and the politics of the climate crisis [file sj-pdf-1-pus-10.1177_09636625221095353.pdf]

Supplemental Material

"It's just a Band-Aid!": Public Engagement with Geoengineering and the Politics of the Climate Crisis

António Carvalho

(Centre for Social Studies, University of Coimbra, Portugal)

Mariana Riquito

(Centre for Social Studies, University of Coimbra, Portugal; Amsterdam Institute for Social Science Research, University of Amsterdam, Netherlands)

Contents

This is the Power Point presentation we used to introduce participants to the topic of Geoengineering. It started by briefly contextualizing the climate crisis, then presented the definition of geoengineering and its two main techniques – Carbon Dioxide Removal (CDR) and Solar Radiation Management (SRM), resorting to examples of on-going projects - David Keith's SCoPEx, the Ocean Iron Fertilization Program of the GEOMAR Helmholtz Centre for Ocean Research, and Climeworks' CDR project.

Grupo de Discussão sobre Alterações Climáticas: Desafios, Soluções, Dilemas

Projeto TROPO – Ontologias do Antropoceno em Portugal: movimentos sociais, políticas públicas e tecnologias emergentes

ces
Centro de Estudos Sociais
Universidade de Coimbra
Centre for Social Studies
University of Coimbra

· U C ·

Organização das Nações Unidas para a Educação a Ciência e a Cultura
Universidade de Coimbra – Alta e Sofia inscrita na Lista do Património Mundial em 2013

O Desafio Climático

“As alterações climáticas são a questão decisiva do nosso século e encontramo-nos num momento chave.”

Nações Unidas

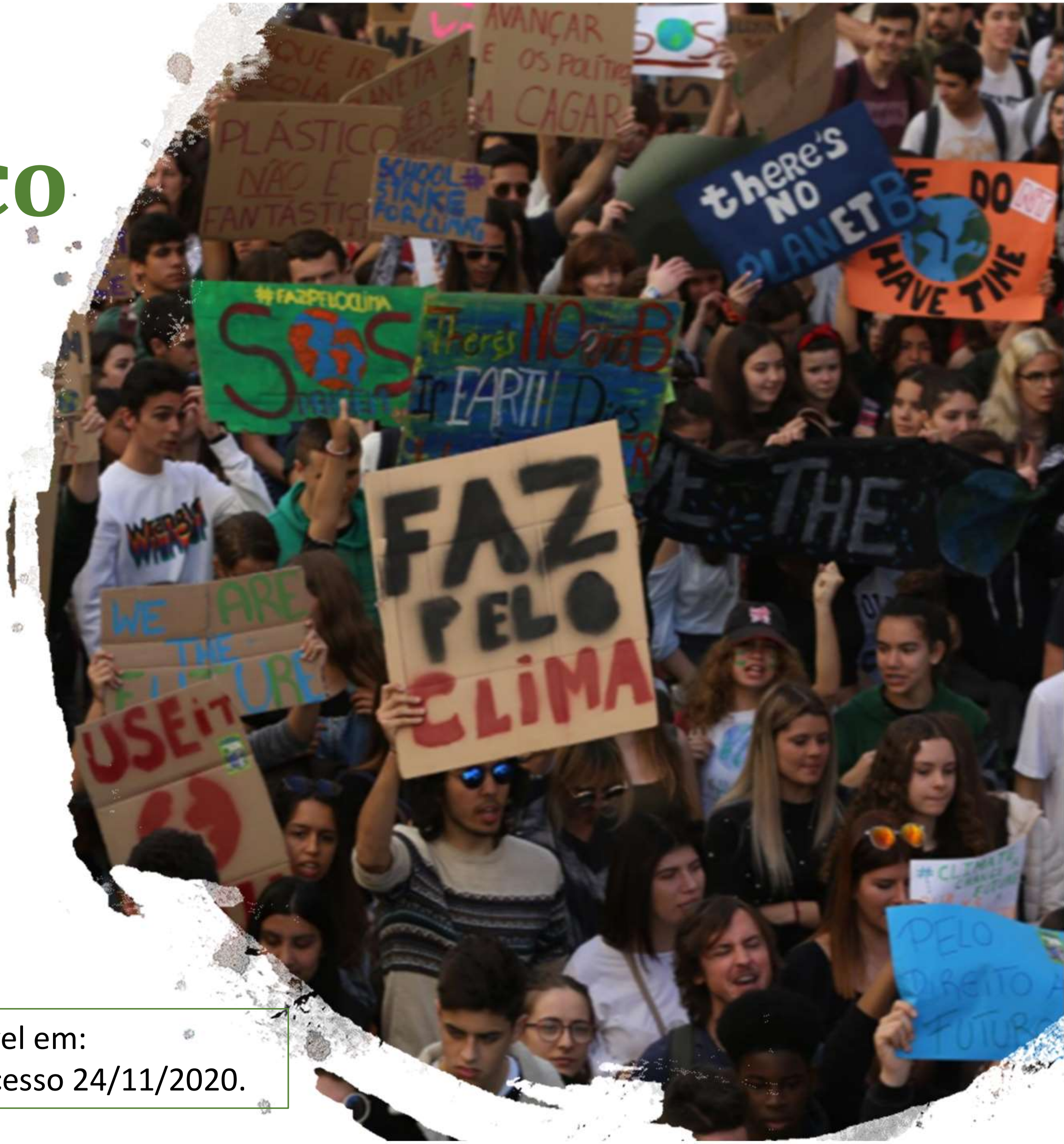

Fonte: United Nations (s. d.) “Climate Change”. Disponível em: https://www.un.org/en/sections/issues-depth/climate-change/ . Acesso 24/11/2020.

Respostas ao Desafio Climático

Mitigação

Medidas que visam combater as causas e minimizar os impactos das alterações climáticas

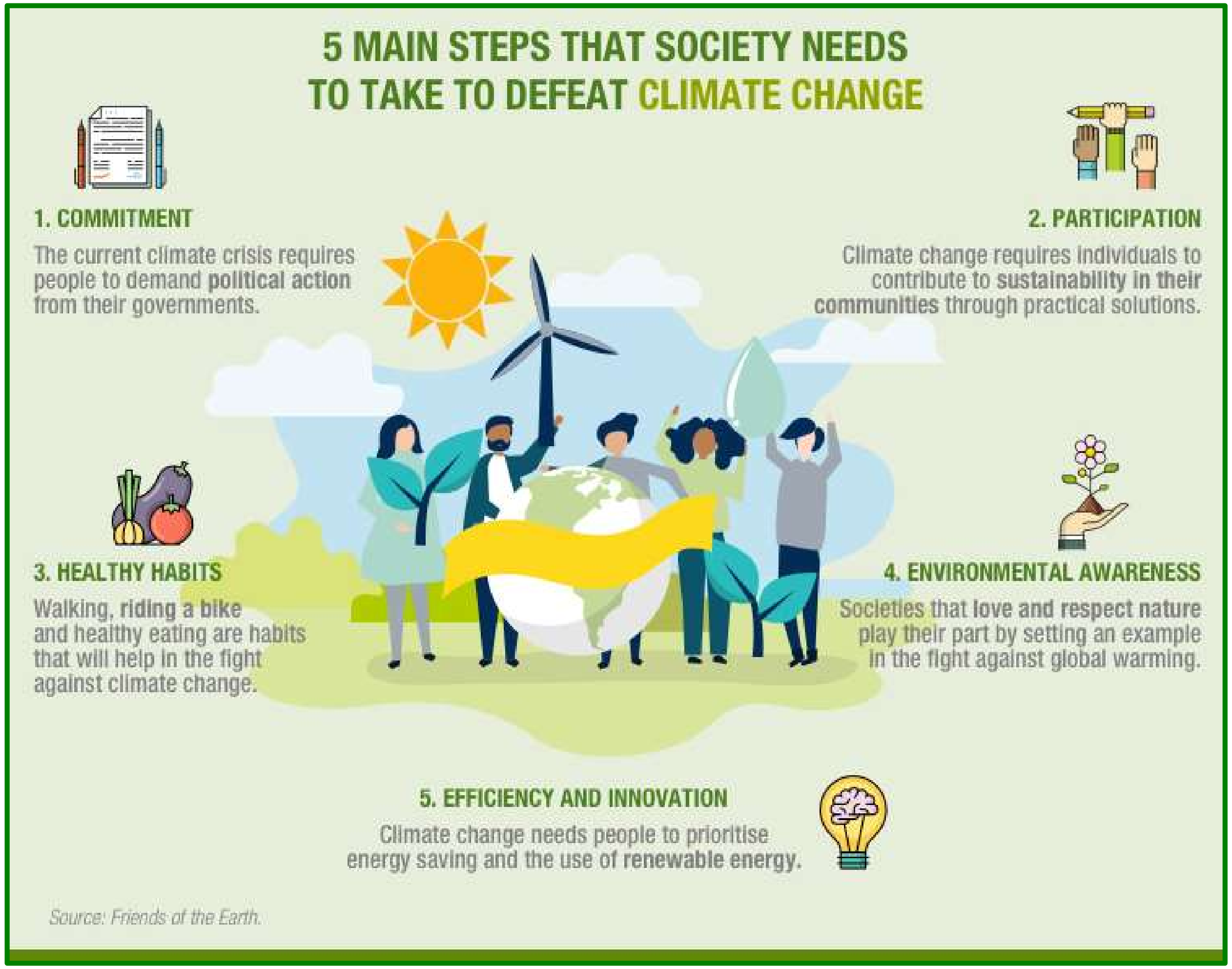

Adaptação

Medidas que visam reduzir as consequências negativas das alterações climáticas, adaptando as estruturas existentes

Geoengenharia: uma possível solução?

A geoengenharia refere-se ao conjunto de métodos e de tecnologias que visam modificar o sistema climático, de forma intencional e em larga escala, para diminuir os impactos nefastos das alterações climáticas.

Fonte: Keith, David (2001) "Geoengineering" *Nature*. 409(6818), 420

Dois Tipos de Técnicas de Geoengenharia

Gestão da Radiação Solar (SRM)

Objetivo?

Reduzir a quantidade de energia solar absorvida pelo sistema climático

Remoção de Dióxido de Carbono (CDR)

Objetivo?

Remover o CO2 diretamente da atmosfera

Fonte: Relatório da Reunião de peritos do IPCC sobre Geoengenharia, em Lima, Peru, nos dias 20-22 Junho de 2011

Atuais Projetos de Gestão da Radiação Solar

Experiência de Perturbação Controlada Estratosférica (SCOPEX),

Projeto de David Keith & Frank Keutsch (Harvard)

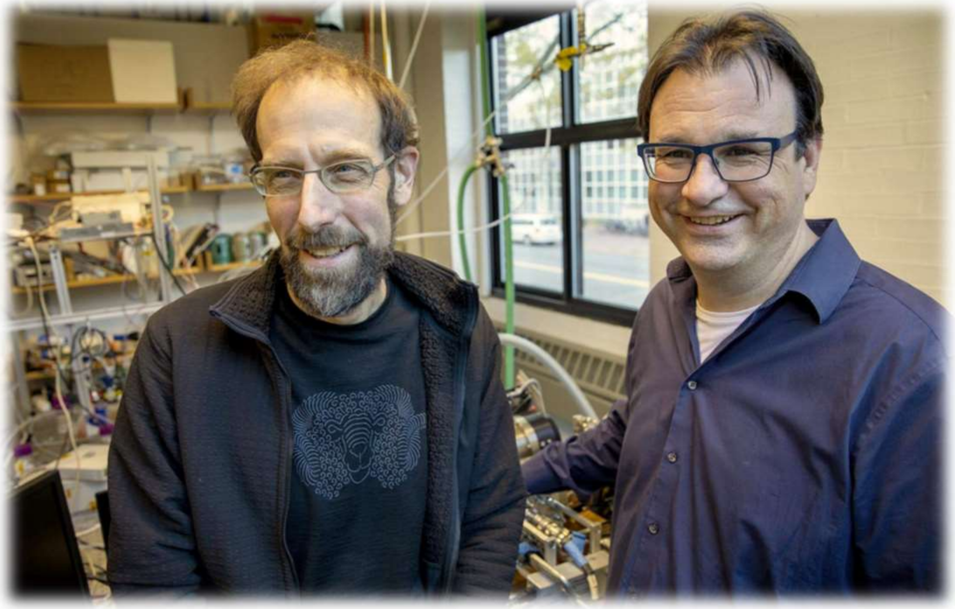

David Keith & Frank Keutsch

Este projeto consiste na libertação de partículas de carbonato de cálcio na estratosfera, de modo a refletir a luz solar e, consequentemente, arrefecer o planeta.

Atuais Projetos de Gestão da Radiação Solar

Experiência de Perturbação Controlada Estratosférica (SCOPEX)

Projeto de David Keith & Frank Keutsch

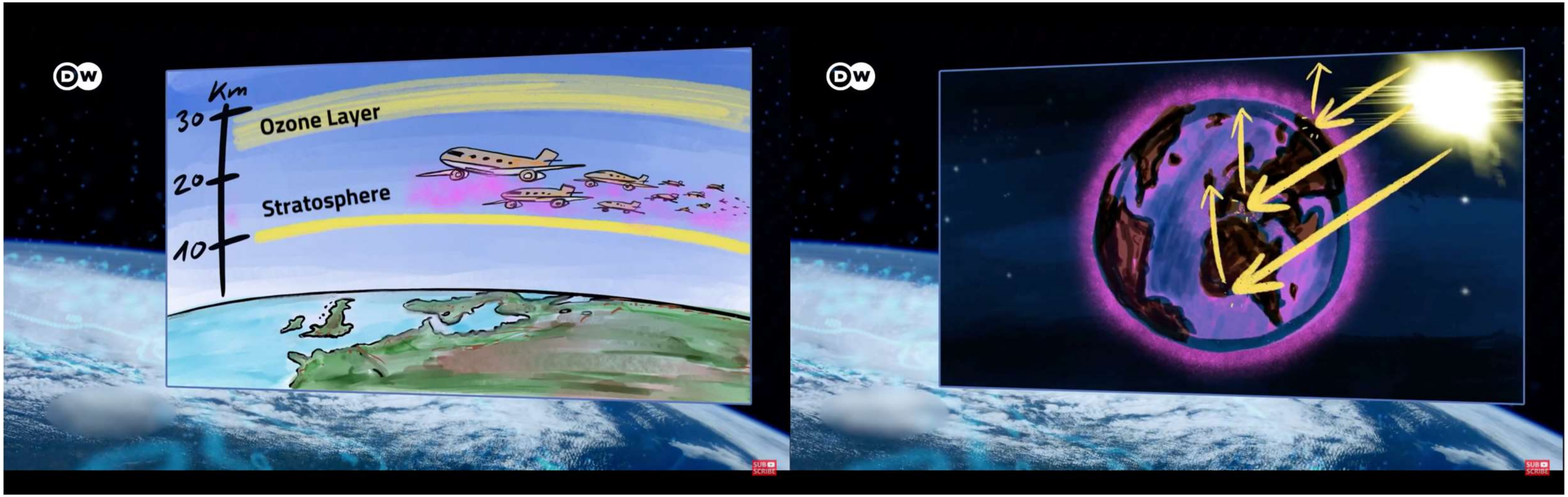

Atuais Projetos de Remoção do Dióxido de Carbono

Ocean Iron Fertilization

Projeto do Centro de Pesquisas Oceânicas Helmholtz (GEOMAR)

O Centro de Pesquisas Oceânicas Helmholtz tem vindo a trabalhar em técnicas de aperfeiçoamento artificial dos oceanos.

Uma dessas técnicas é a fertilização dos oceanos, com recurso a pó de ferro, que facilita o desenvolvimento de algas bem como de outras espécies, permitindo “sequestrar” o CO2 nos oceanos.

Estas experiências estão a ser atualmente conduzidas em Lima, no Peru.

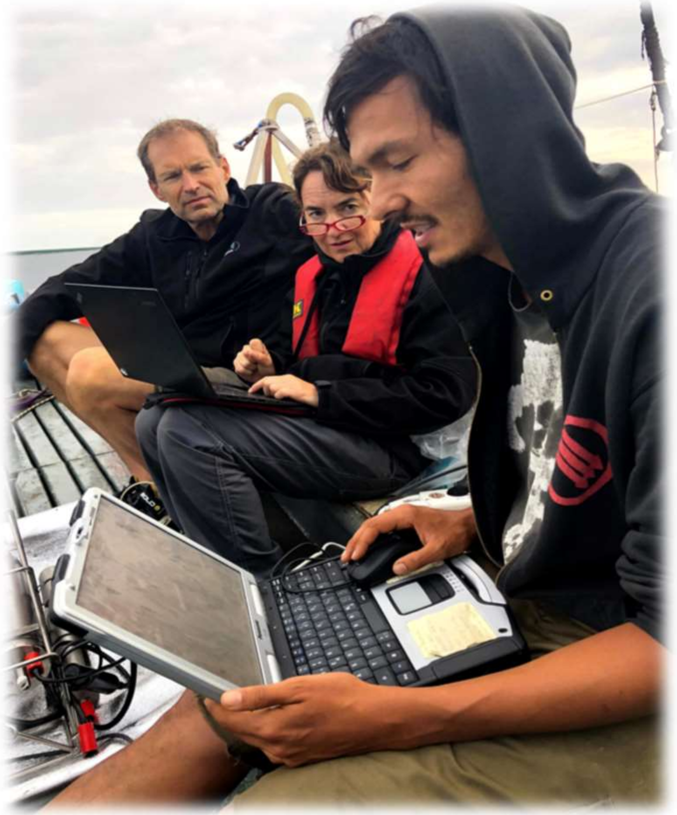

Equipa do GEOMAR

Atuais Projetos de Remoção do Dióxido de Carbono

Ocean Iron Fertilization

Projeto do Centro de Pesquisas Oceânicas Helmholtz (GEOMAR)

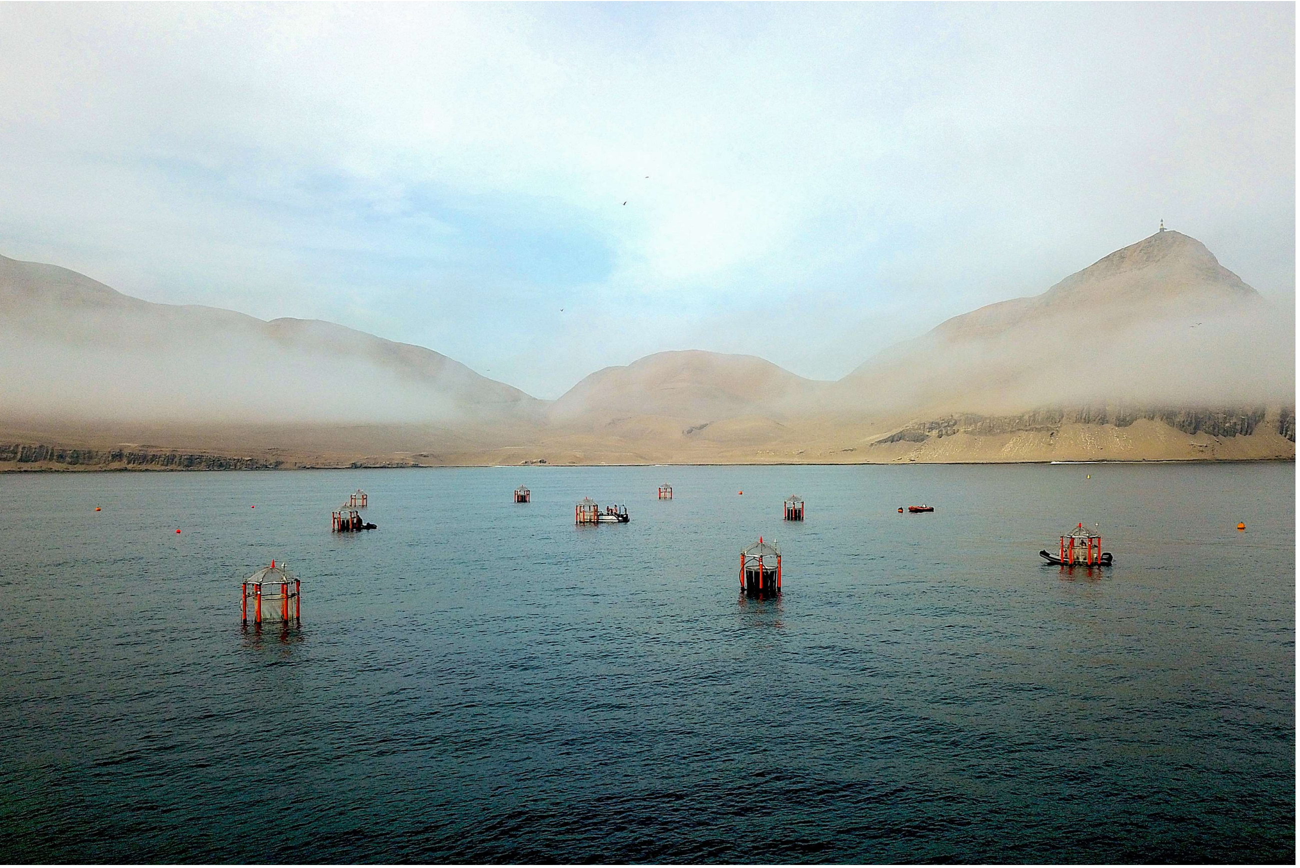

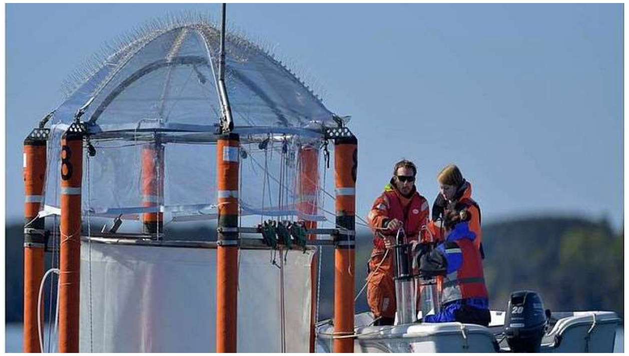

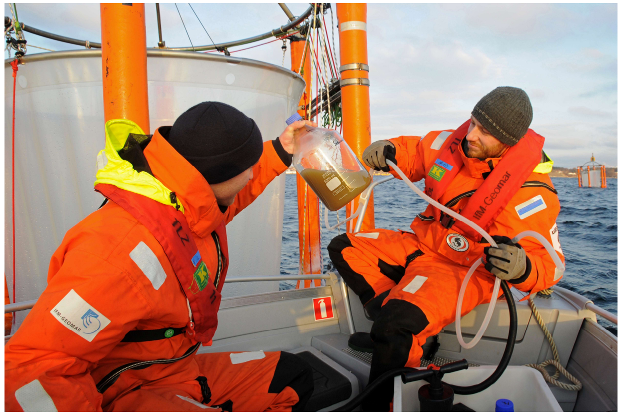

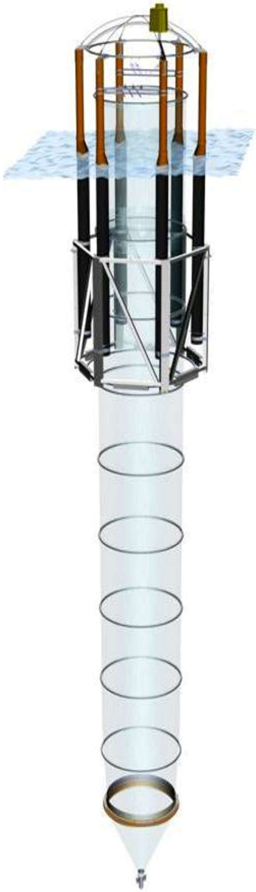

Atuais Projetos de Remoção do Dióxido de Carbono

Captura e Armazenamento Diretos de CO_2

Projeto da Climeworks

A *start-up* suíça *Climeworks* desenvolveu coletores que permitem capturar o dióxido de carbono diretamente do ar.

Uma vez capturado, esse CO_2 pode ser reciclado, usado como matéria-prima, armazenado em formações geológicas, ou até completamente removido do ar.

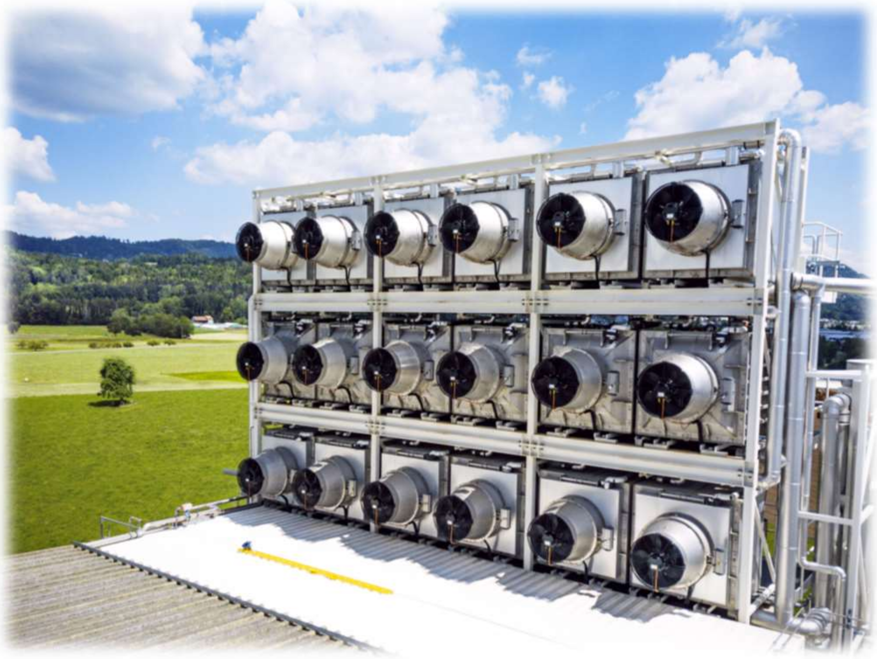

A Climeworks tem atualmente 15 máquinas em operação na Europa.

Atuais Projetos de Remoção do Dióxido de Carbono

Captura e Armazenamento Diretos de CO2

Projeto da Climeworks

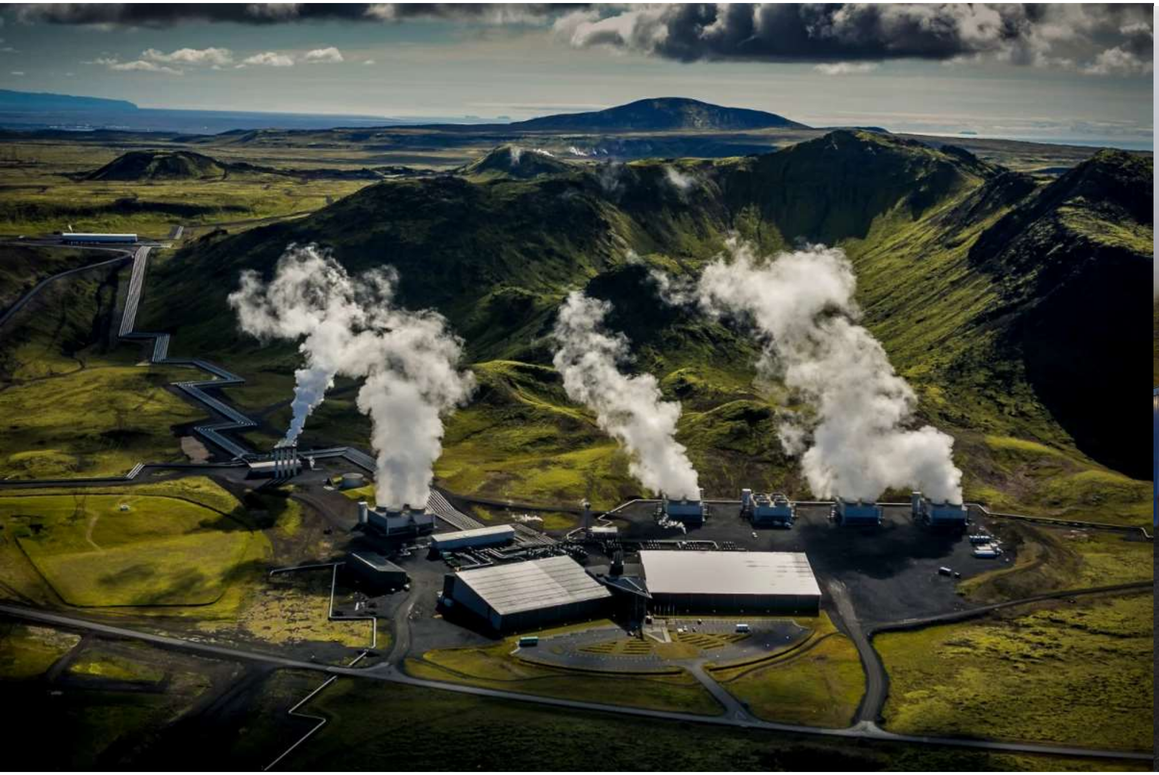

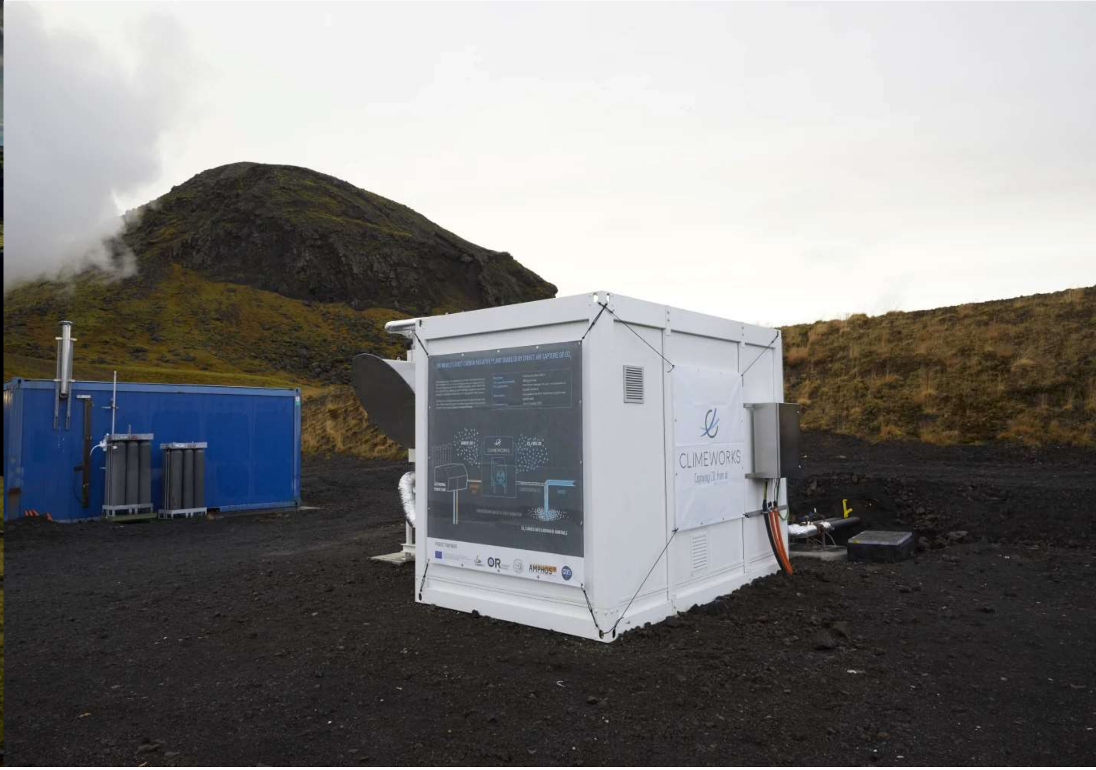

Possíveis Projetos Futuros de Geoengenharia

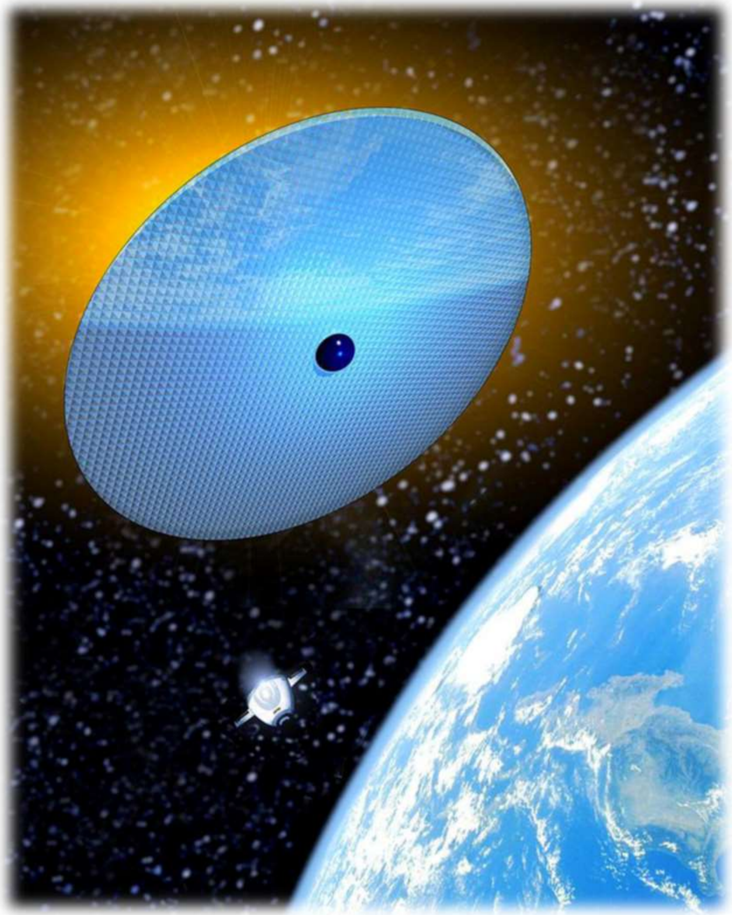

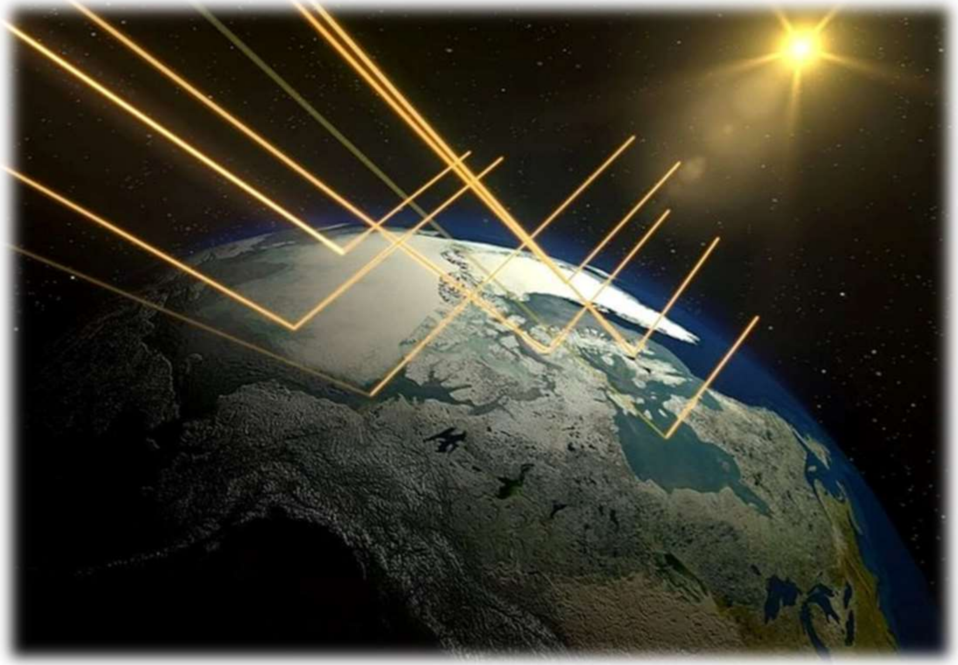

- ❖ Criação de Escudos Solares no Espaço
- ❖ Modificação Genética de Plantas

Discussão de Grupo

Projeto TROPO – Ontologias do Antropoceno em Portugal: movimentos sociais, políticas públicas e tecnologias emergentes

Centro de Estudos Sociais Universidade de Coimbra
Centre for Social Studies University of Coimbra

Fontes das Imagens

- Slide 3: Foto de Miguel Manso, Disponível em https://www.publico.pt/2019/03/20/p3/cronica/planeta-mudar-nao-1865934 (Acesso em 20 de novembro de 2020)

- Slide 4: Gráfico de Friends of the Earth, disponível em https://www.iberdrola.com/sustainability/climate-change-mitigation-and-adaptation (Acesso em 20 de novembro de 2020)

- Slide 7: Foto de Robbin Lubbock/WBUR, disponível emhttps://www.wbur.org/news/2020/07/22/harvard-solar-geoengineering-climate-change (Acesso em 20 de novembro de 2020)

- Slide 8: Screenshots retirados de https://www.youtube.com/watch?v=b1Enrzgrl1w&ab_channel=DWDocumentary (Acesso em 1 de dezembro de 2020)

Fontes das Imagens

- Slides 9 & 10: Fotos de GEOMAR, disponível em https://www.geomar.de/en/news/article?tx_news_pi1%5baction%5d=detail&tx_news_pi1%5bcontroller%5d=News&tx_news_pi1%5bactbackPid%5d=12123&tx_news_pi1%5bbackPid%5d=12123&tx_news_pi1%5bnews%5d=7338 (Acesso em 20 de novembro de 2020)

- Slides 11 & 12: Fotos de Climeworks, disponível em https://climeworks.com/ (Acesso em 1 de dezembro de 2020)

- Slide 13: Imagem de Victor Habbik, disponível em https://www.forbes.com/sites/startswithabang/2020/01/04/ask-ethan-could-we-just-build-a-space-shade-to-counteract-global-warming/?sh=1437b50a43bc (Acesso em 1 de dezembro de 2020) // Foto de NASA, disponível em https://www.treehugger.com/reasons-why-arctic-sea-ice-matters-4868655 (Acesso em 1 de dezembro de 2020)
